# An ingroup disadvantage in recognizing micro-expressions

**DOI:** 10.3389/fpsyg.2022.1050068

**Published:** 2022-11-25

**Authors:** Qi Wu, Kunling Peng, Yanni Xie, Yeying Lai, Xuanchen Liu, Ziwei Zhao

**Affiliations:** ^1^Department of Psychology, School of Educational Science, Hunan Normal University, Changsha, China; ^2^Cognition and Human Behavior Key Laboratory of Hunan Province, Hunan Normal University, Changsha, China

**Keywords:** micro-expression, macro-expression, recognition, emotion perception, intergroup bias, Ingroup advantage, ingroup disadvantage

## Abstract

Micro-expression is a fleeting facial expression of emotion that usually occurs in high-stake situations and reveals the true emotion that a person tries to conceal. Due to its unique nature, recognizing micro-expression has great applications for fields like law enforcement, medical treatment, and national security. However, the psychological mechanism of micro-expression recognition is still poorly understood. In the present research, we sought to expand upon previous research to investigate whether the group membership of the expresser influences the recognition process of micro-expressions. By conducting two behavioral studies, we found that contrary to the widespread ingroup advantage found in macro-expression recognition, there was a robust ingroup disadvantage in micro-expression recognition instead. Specifically, in Study 1A and 1B, we found that participants were more accurate at recognizing the intense and subtle micro-expressions of their racial outgroups than those micro-expressions of their racial ingroups, and neither the training experience nor the duration of micro-expressions moderated this ingroup disadvantage. In Study 2A and 2B, we further found that mere social categorization alone was sufficient to elicit the ingroup disadvantage for the recognition of intense and subtle micro-expressions, and such an effect was also unaffected by the duration of micro-expressions. These results suggest that individuals spontaneously employ the social category information of others to recognize micro-expressions, and the ingroup disadvantage in micro-expression stems partly from motivated differential processing of ingroup micro-expressions.

## Introduction

Facial expressions are of paramount importance in our social world. We are moved and motivated by people’s smiles and grimaces, which are the unique signals of the emotions and intentions of others. Our daily interactions depend on the accurate interpretation of these social signals (Niedenthal and Brauer, 2012). However, not all our emotions are spontaneously expressed by the face. To achieve our personal goals, we all need to hide, disguise, or inhibit our true emotions in a number of situations (Ekman, 1971). Fortunately, these inhibited emotions may still leak, which results in a unique form of facial expression, that is, the micro-expressions (e.g., Yan et al., 2013; Li et al., 2022). Micro-expression is an extremely quick facial expression of emotion that lasts less than 0.5 s (Matsumoto and Hwang, 2011, 2018; Yan et al., 2013). This kind of expression is more likely to leak under high-stakes situations, especially when someone is trying to hide or disguise something important (e.g., the attack intention; Ekman, 2009). Due to its unique nature, recognizing micro-expression has great applications in fields like law enforcement, medical treatment, education, and national security (e.g., Russell et al., 2008; Weinberger, 2010; Matsumoto and Hwang, 2011, 2018; Stewart and Svetieva, 2021). Prior studies have suggested that the micro-expression recognition process is influenced by factors like profession, personality, and social or emotional context (e.g., Matsumoto et al., 2000; Frank et al., 2009; Hurley et al., 2014; Svetieva and Frank, 2016; Demetrioff et al., 2017; Felisberti, 2018; Zhang et al., 2020). In the present research, we sought to expand upon previous research to investigate whether the recognition of micro-expressions is influenced by the group membership of the expresser.

Group membership, particularly the distinction between “us” (ingroup) and “them” (outgroup), is fundamentally important for human interactions (Hewstone et al., 2002). Across a variety of scenarios, people tend to show a systematic tendency to favor the member of their ingroup over the member of outgroup (i.e., ingroup favoritism) based on real-world salient groupings like race, ethnicity, religiosity, and political affiliation (e.g., Hewstone et al., 2002; Fu et al., 2012). Such a favoritism toward ingroup members has also been demonstrated in the laboratory by using trivial groupings (i.e., the minimal group paradigm; e.g., Tajfel et al., 1971; Makhanova et al., 2022). More importantly, researchers have found that the group membership has also influenced our emotion communications (Elfenbein and Ambady, 2002; Elfenbein et al., 2007). Specifically, researchers found that people are more accurate at recognizing macro-expressions (i.e., the typically expressed facial expressions that last for 0.5–4 s when emotions occur; Matsumoto and Hwang, 2011, 2018) of individuals belonging to their ingroup (e.g., sharing the same culture, race, ethnic group, religion, or fan membership) compared to macro-expressions displayed by people from outgroup (Elfenbein and Ambady, 2002; Elfenbein et al., 2007; Tuminello and Davidson, 2011; Huang and Han, 2014; Zhang et al., 2015; Friesen et al., 2019; Kommattam et al., 2019; Handley et al., 2021; Fang et al., 2022; Hess et al., 2022). This ingroup advantage can be partially explained by the dialect theory, which proposed that there are subtle differences in the expressions displayed by members of different groups (Elfenbein et al., 2007). However, some researchers also found that mere social categorization alone (i.e., the minimal group) was sufficient to elicit the ingroup advantage in macro-expression recognition (e.g., Thibault et al., 2006; Young and Hugenberg, 2010; Young and Wilson, 2018). These researches suggest that we are motivated to decode or attend to the facial expressions of ingroup members in different ways (Elfenbein, 2015; Hess et al., 2022), and this ingroup advantage may already exist in the early processing stage of facial expression recognitions (Gamond et al., 2017).

Can the group membership also affect the recognition of micro-expressions? As far as we know, no previous studies have directly addressed this issue. Answers to this question will deepen our understanding of the micro-expression recognition process which provides the necessary knowledge for developing more efficient micro-expression recognition training programs (Matsumoto and Hwang, 2011; Hurley et al., 2014; Döllinger et al., 2021) or building more valid micro-expression databases (Yan et al., 2014; Yap et al., 2020; Li et al., 2022). In fact, previous studies have found that micro-expressions and macro-expressions are usually identical in their appearances (Ekman, 2003; Matsumoto and Hwang, 2011, 2018; Yan et al., 2013) and there are some similarities between the recognition of micro-expressions and the recognition of macro-expressions. For example, researchers have found that both the recognitions of micro-expression and macro-expression are affected by factors like age, personality, profession, training experience, mental disorders of perceivers, and the emotional context of facial expressions (Matsumoto et al., 2000; Frank et al., 2009; Hurley, 2012; Hurley et al., 2014; Svetieva and Frank, 2016; Demetrioff et al., 2017; Zhu et al., 2017; Zhang et al., 2020; Döllinger et al., 2021; Fan et al., 2022). However, previous studies also suggest that there might be fundamental differences in the neuropsychological basis for micro-expression recognition and macro-expression recognition. For example, although the facial mimicry has been demonstrated to be an effective cue in macro-expression recognition, enhancing facial mimicry that occurred during emotion perception process has been demonstrated to impair the recognition accuracy of micro-expressions (Wu et al., 2016a; Zeng et al., 2018). Researchers also found that the EEG/ERPs mechanisms are different for the recognition of micro-expressions and the recognition of macro-expressions, and these differences are mainly located in the inferior temporal gyrus and front lobe (Shen et al., 2016). More importantly, one recent study showed that while the ancient neuropeptide oxytocin has been demonstrated to have a facilitating role in the recognition of macro-expressions, intranasal oxytocin administration was found to disrupt the recognition of micro-expressions (Wu et al., 2022). Given that researchers also found that the expressions of ingroup members are more likely to elicit facial mimicry from perceivers (e.g., Hess and Fischer, 2014; Hess et al., 2022) and the neuropeptide oxytocin are more likely to affect the social tendency toward ingroup members (e.g., De Dreu et al., 2011; McClung et al., 2018), these previous studies suggest that the facial mimicry generated during emotion perception and the endogenous oxytocin generated in our brain are more likely to disrupt the recognition of ingroup micro-expressions, which may further create an ingroup disadvantage in micro-expression recognition. Therefore, in the present study, we hypothesized that the group membership of the expresser would also affect the recognition of micro-expressions and there would be an ingroup disadvantage for micro-expression recognitions (i.e., the overall recognition accuracy of the micro-expressions of ingroup members would be lower than the overall recognition accuracy of the micro-expressions of outgroup members).

To test this hypothesis, we conducted two behavioral studies in the current research. Specifically, we first investigated whether there is an ingroup disadvantage for micro-expression recognition in real-world social groups (i.e., race) in Study 1A^1^ and 1B. In Study 2A and 2B, we further investigated whether mere social categorization alone (i.e., the minimal group) is sufficient to elicit the ingroup disadvantage in micro-expression recognition. Furthermore, prior researches showed that the accuracy of micro-expression recognition may be affected by the duration of micro-expression (Shen et al., 2012; Wu et al., 2016a, 2022; Zeng et al., 2018). Therefore, following previous studies (Felisberti, 2018; Zeng et al., 2018; Wu et al., 2022), we employed two different settings of duration (i.e., 100 ms and 333 ms) in the present research.

## Study 1

As one of the salient cues for real-world groupings, information about one’s race has been shown to greatly affect the recognition of macro-expressions (e.g., Elfenbein and Ambady, 2002; Elfenbein et al., 2007; Elfenbein, 2015; Zhang et al., 2015; Friesen et al., 2019; Fang et al., 2022). Therefore, in Study 1, we tested our hypothesis by employing real-world racial groups at first (i.e., White vs. Asian). Specifically, given that the intensity of the facial actions of micro-expressions may affect the recognition accuracy of micro-expressions (Wu et al., 2016a, 2022; Zeng et al., 2018), in Study 1A, we investigated whether there is an ingroup disadvantage for micro-expression recognition under racial group situations by employing micro-expressions that are high in intensity (intense micro-expressions; Wu et al., 2016a, 2022; Zeng et al., 2018). In Study 1B, we further tested our hypothesis by employing micro-expressions that have low intensities (subtle micro-expressions; Wu et al., 2016a, 2022; Zeng et al., 2018) under race-based grouping situations. Considering that previous studies have also suggested that the recognition accuracy of micro-expressions can be significantly improved after receiving the training of Micro Expression Training Tool (METT; Russell et al., 2008; Ekman, 2009; Frank et al., 2009; Matsumoto and Hwang, 2011; Hurley, 2012; Hurley et al., 2014), in Study 1A and 1B we also explored whether trainings in micro-expression recognition can moderate the ingroup disadvantage effect in micro-expression recognition.

### Study 1A

#### Participants and design

The required sample size (1 – β = 0.85, α = 0.05) was estimated by using G*Power (Faul et al., 2009). Given the current experimental design and using *f* = 0.2148 as the expected effect size (the mean effect size in social psychology; Richard et al., 2003), we estimated a sample size of 84. Finally, a total of 84 Chinese college students (44 females and 40 males; *M*_age_ = 19.52, *SD* = 1.86, aged 18–25 years; training condition: *n* = 42; control condition: *n* = 42) were recruited through advertising on campus.

A 2 (target group: ingroup, outgroup) × 2 (duration: 100 ms. 333 ms) × 2 (METT training: training, control) × 2 (test: pre-test, post-test) mixed design was used. The METT training was the between-subjects factor. The target group, the duration, and the test were the within-subjects factors.

#### Facial stimuli

Facial expression images of 47 models (24 Whites and 23 Asians) were selected from the BU-3DFE database (Yin et al., 2006). This database contains facial expression images from 100 models (aged 18–70 years) of a variety of races, including White, Black, Asian, etc. For each model, one facial image of his/her neutral expression and facial images of his/her six universal facial expressions (i.e., happiness, disgust, fear, angry, surprise, and sadness) are included in the database, and each of the six universal facial expressions includes four levels of intensity (i.e., low, middle, high, and very high).

As for these 47 selected models from BU-3DFE database, facial images of their neutral expressions and the six universal facial expressions that were rated to be “very high” in intensity were employed. In addition, the neutral face and the six universal facial expressions of one additional Asian model from NimStim database (Tottenham et al., 2009) were also employed (only in the pilot study, not in the formal study). All employed facial images were then rescaled to a unified size (512 × 512 pixels) and were normalized by converting them to 8-bit grey scale images and normalizing their grey values to grand mean (Liu and Chen, 2012).

To select the appropriate White and Asian models for the formal study and to match the recognition accuracy of macro-expressions for these models, a pilot study was carried out. In the pilot study, 30 Chinese college students were asked to recognize these normalized facial expressions (excluding the neutral faces) of the 48 selected models (i.e., 47 models from the BU-3DFE database and one Asian model from the NimStim database). These expressions were presented as macro-expressions. Specifically, the facial expressions were displayed on the screen for 1 s, then participants were asked to recognize the presented facial expressions at self-paced speed (stimuli remained on the screen until the response was made) and as accurate as possible.

Based on the accuracy data of pilot study, 24 models (half Whites and half Asians) from the BU-3DFE database were selected. These selected 24 models were further divided into four different model groups. Specifically, the models from each race were further divided into two model groups (i.e., W_1_ and W_2_ for White models, A_1_ and A_2_ for Asian models). Therefore, there were six models in each group, and half of the models were females, while the other half were males. Then, the normalized facial images of W_1_ were merged with the normalized facial images of A_1_ to form the stimuli set A, and the normalized facial images of W_2_ were merged with the normalized facial images of A_2_ to form the stimuli set B. A 2 (race: White, Asian) × 2 (stimuli set: A, B) repeated-measures analysis of variance (ANOVA) on the accuracy data of these 24 selected models showed that there were no significant differences in recognition accuracy among the macro-expressions of White models and the macro-expressions of Asian models from different stimuli sets (all the main effects and the race × stimuli set interaction were not significant, *F*s < 0.12, *p*s > 0.73). Therefore, the stimuli set A and the stimuli set B were employed as the facial stimuli for micro-expression recognition tasks in Study 1A. In sum, 144 images of facial expressions and 24 images of neutral faces were employed.

#### Experimental tasks and procedures

There were three stages in Study 1A, including the pre-test, the training, and the post-test. After entering the lab and providing their informed consent, participants were randomly assigned to one of the two METT training conditions (i.e., the training condition and the control condition). Then all participants had to complete the pre-test of micro-expression recognition task. After that, participants in the training condition were asked to receive the METT training, and participants in the control conditions were asked to rest for 25 min (approximately the same amount of time the METT training would take). Participants in the training condition were asked to rest for 5 min after the training of METT and then they were asked to finish the post-test of micro-expression recognition task. Participants in the control condition were directly asked to finish the post-test of micro-expression recognition task when their rest was over.

##### Micro-expression recognition tasks

In Study 1A, participants had to complete two micro-expression recognition tasks (i.e., pre-test and post-test). In each micro-expression recognition task, only one stimuli set (i.e., stimuli set A or B) was presented (the combination order of stimuli sets and micro-expression recognition tasks was counterbalanced across participants). For models of each stimuli set, they were randomly assigned to one of the two duration conditions (100 ms or 333 ms) and all the six universal micro-expressions (i.e., happiness, disgust, fear, angry, surprise, and sadness) of the models were presented according to their assigned condition. The micro-expressions of each model were presented only once. Therefore, in each micro-expression recognition task, there were three White and three Asian models in each duration condition (72 trials in total in each task). Since all our participants were Chinese, the stimuli of ingroup member were constituted by the micro-expressions of Asian models, while the stimuli of outgroup members were constituted by the micro-expressions of White models.

**Figure 1:**
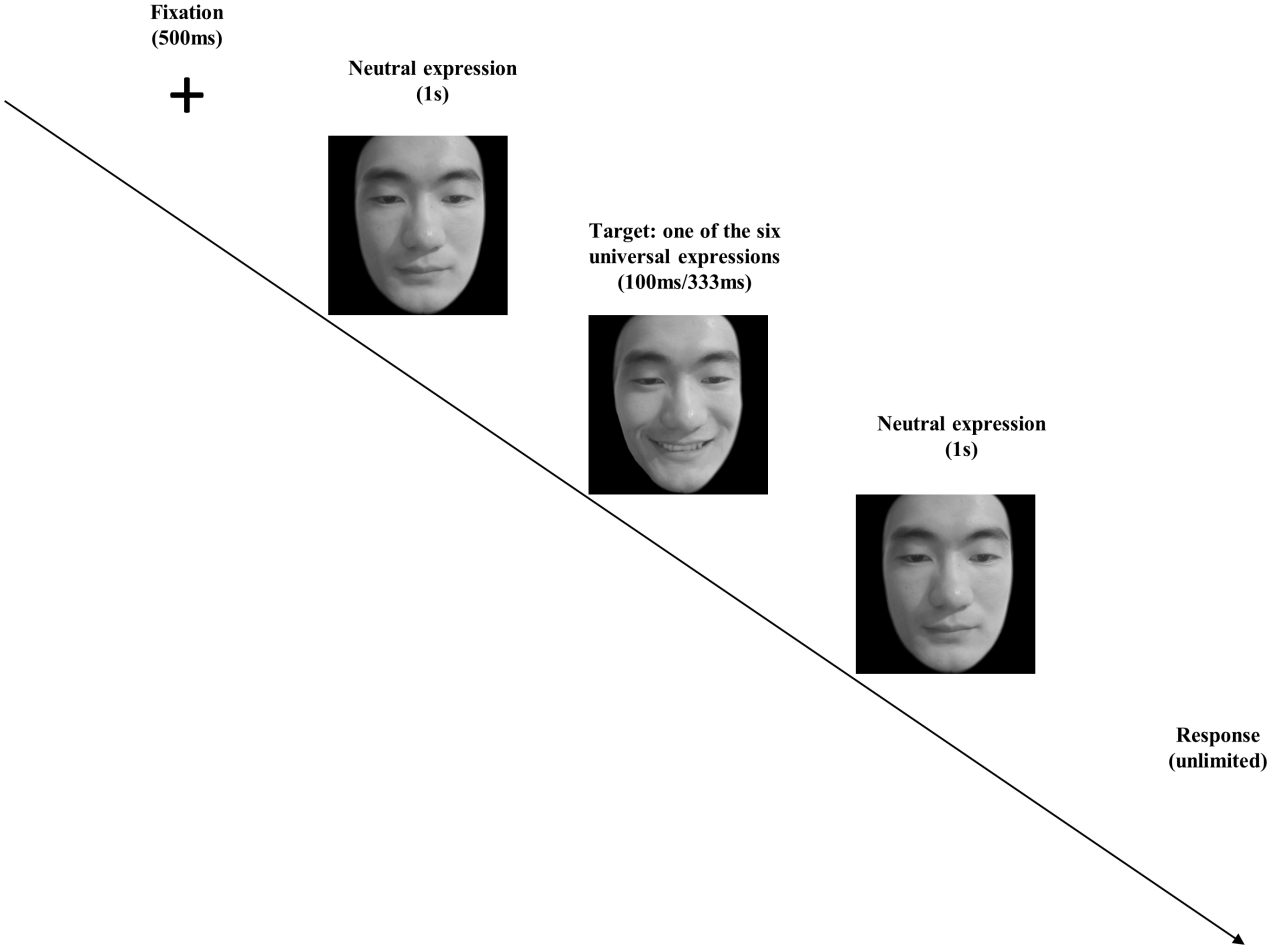
The procedure of the micro-expression recognition task in Study 1A. Note that we employ the facial images of the second author for illustration.

In both the pre-test and post-test of micro-expression recognition tasks, we employed the well-accepted paradigm of Japanese and Caucasian Brief Affect Recognition Test (JACBART; e.g., Matsumoto et al., 2000; Hurley, 2012; Hurley et al., 2014; Wu et al., 2016a, 2022; Demetrioff et al., 2017; Zhang et al., 2020; Döllinger et al., 2021; Fan et al., 2022) to present micro-expressions. In this paradigm, a fixation cross was presented for 500 ms at first, after that a facial expression image was presented according to its assigned duration (i.e., 100 ms or 333 ms in Study 1A), which was sandwiched in between two 1 s presentations of the same expresser’s neutral face (see Figure 1). Then, participants were instructed to recognize the micro-expression that just flashed on the screen at self-paced speed and as accurate as possible (respond by choosing one out of seven options, including happiness, disgust, fear, angry, surprise, sadness, and an option of “none of the above”). The presentation of the stimulus was completely randomized. The recognition accuracy was recorded for micro-expression recognition tasks.

##### Micro-expression recognition training

In Study 1A, the METT training package (Ekman, 2002) was employed to explore the moderating effect of training experience. The METT is a self-instructional training program comprised of five sections: pre-and post-tests, training, practice, and review. In Study 1A, we only utilized the three sections of this training package. Participants were asked to finish the training, practice, and review sections of METT in a sequential order. In the training and review sections, participants were taught to distinguish between commonly confused facial expressions of both Whites and Asians by watching videos. Participants were instructed to practice the acquired recognition skills with 28 micro-expressions of male and female expressers of White or Asian models in the practice section. For each participant, the METT training was finished under the supervision of experimenter. The micro-expressions and expression videos in METT were taken from the Japanese and Caucasian Brief Affect Recognition Test (Matsumoto et al., 2000; Ekman, 2002). None of these materials were presented in the micro-expression recognition tasks of Study 1A.

#### Results and discussion

**Figure 2:**
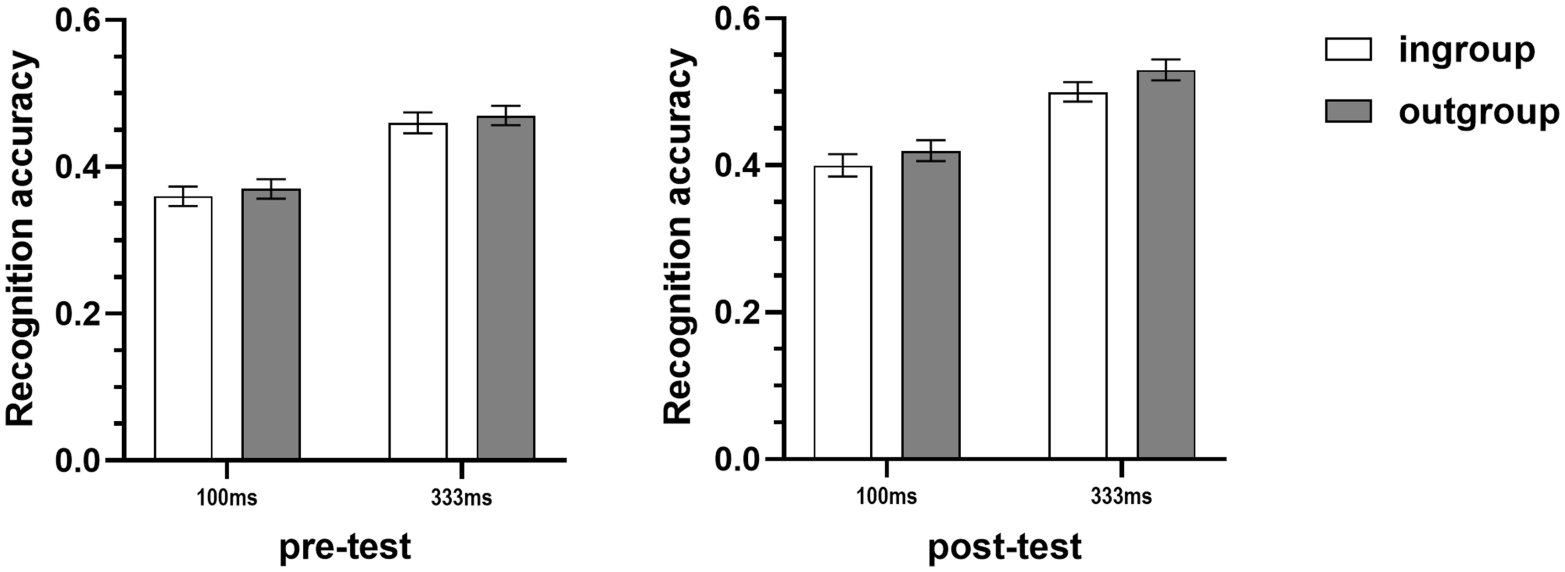
Mean recognition accuracies of micro-expressions of ingroup and outgroup members collapsed across two METT training conditions (Study 1A). Error bars represent standard errors.

The recognition accuracies of micro-expression recognition tasks were subjected to a 2 (target group) × 2 (duration) × 2 (METT training) × 2 (test) mixed-model ANOVA. The results showed that the main effect of target group was significant, the recognition accuracy of micro-expressions of outgroup members (*M* = 0.45, *SD* = 0.09) was higher than the recognition accuracy of micro-expressions of ingroup members (*M* = 0.43, *SD* = 0.09) (see Table 1 and Figure 2). In addition, the results also showed that neither the duration of micro-expression nor the METT training moderated this ingroup disadvantage, and this ingroup disadvantage effect was also consistent for both the pre-test and post-test of micro-expression recognition task (see Table 1). Furthermore, the results also showed that the main effect of METT training and the METT training × test interaction were significant. Further simple effects analysis showed that in the pre-test of micro-expression recognition task, no significant differences in recognition accuracies were found between the training condition (*M* = 0.43, *SD* = 0.08) and the control condition (*M* = 0.40, *SD* = 0.11), *F*(1,82) = 1.36, *p* = 0.25, η_p_^2^ = 0.02; but the METT training significantly improved the recognition accuracy for participants in the training condition, their recognition accuracy (*M* = 0.49, *SD* = 0.09) became significantly higher than that of control condition (*M* = 0.43, *SD* = 0.10) in the post-test of micro-expression recognition task [*F*(1,82) = 9.73, *p* = 0.003, η_p_^2^ = 0.11], which indicates that the METT training was effective. The results also showed that all the other two-way, three-way, and four-way interactions were not significant (see Table 1).

**Table 1:** The results of mixed model ANOVA in Study 1A.

Effect	*F*	*p*	η_p_^2^
Target group	6.34	0.01*	0.07
Duration	156.70	<0.001***	0.66
METT training	5.84	0.04*	0.05
test	21.97	<0.001***	0.21
Target group × duration	0.05	0.83	0.001
Target group × METT training	0.28	0.6	0.003
Target group × test	2.26	0.14	0.03
Duration × METT training	0.09	0.77	0.001
Duration × test	1.06	0.31	0.01
METT training × test	4.49	0.04*	0.05
Target group × duration × METT training	3.4	0.07	0.04
Target group × duration × test	0.5	0.48	0.01
Target group × METT training × test	0.003	0.96	<0.001
Duration × METT training × test	3.24	0.08	0.04
Target group × duration × METT training × test	0.22	0.64	0.003

*p < 0.05, ***p < 0.001.

In sum, by controlling the differences in macro-expression recognition accuracy between Asian and White models, in Study 1A, we found that participants were more accurate at recognizing the micro-expressions of their racial outgroups rather than the micro-expressions of their racial ingroups. These results suggest that an ingroup disadvantage may exist in micro-expression recognition, which is consistent with our hypothesis. It should be noted that in Study 1A, we had only investigated the effects of expresser’s group membership on the recognition of intense micro-expressions. However, it has been shown that micro-expressions are usually very low in intensity (i.e., subtle micro-expressions; Porter and ten Brinke, 2008; Wu et al., 2016a, 2022; Yan et al., 2013). Therefore, in Study 1B, we further investigated the effects of expresser’s group membership on the recognition of subtle micro-expressions.

### Study 1B

#### Participants and design

The required sample size was calculated with G*Power based on the same parameters of Study 1A. A sample size of 84 participants was estimated. Finally, a total of 84 Chinese college students (42 females and 42 males; *M*_age_ = 20.77, *SD* = 1.31, aged 18–25 years; training condition: *n* = 42; control condition: *n* = 42) were recruited through advertising on campus.

A 2 (target group: ingroup, outgroup) × 2 (duration: 100 ms. 333 ms) × 2 (METT training: training, control) × 2 (test: pre-test, post-test) mixed design was used. The METT training was the between-subjects factor. The target group, the duration, and the test were the within-subjects factors.

#### Materials and procedure

Study 1B employed the same materials, experimental tasks, and procedure of Study 1A, except that the facial expressions in Study 1B was low in intensity. Specifically, the employed facial expressions of Study 1B were also selected from the 12 White or Asian models of BU-3DFE database and were normalized as in Study 1A, except that we only employed the facial expressions that were rated to be “low” in intensity (Yin et al., 2006). The results of another pilot study (*n* = 30; in this pilot study, the selected facial stimuli of Study 1B were presented as macro-expressions as in the pilot study of Study 1A) showed that there were no significant differences in recognition accuracy among the subtle macro-expressions of White models and Asian models of different stimuli sets (all the main effects and the race × stimuli set interaction were not significant, *F*s < 0.27, *p*s > 0.62). Since the participants of Study 1B were also Chinese, the stimuli of ingroup member in Study 1B were also constituted by the micro-expressions of Asian models, and the stimuli of outgroup members were also constituted by the micro-expressions of White models.

#### Results and discussion

**Figure 3:**
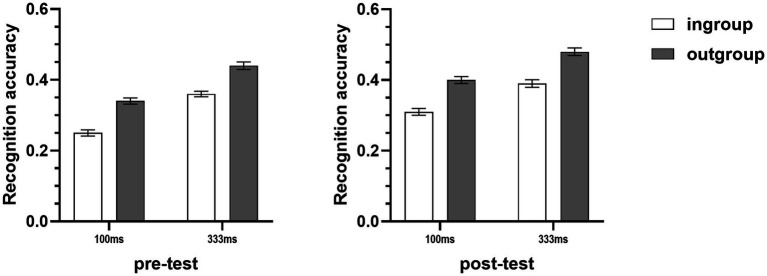
Mean recognition accuracies of micro-expressions of ingroup and outgroup members collapsed across two METT training conditions (Study 1B). Error bars represent standard errors.

The recognition accuracies of micro-expression recognition tasks were subjected to a 2 (target group) × 2 (duration) × 2 (METT training) × 2 (test) mixed-model ANOVA. Consistent with Study 1A, the results showed that there was a significant main effect of target group, participants displayed higher recognition accuracy for micro-expressions of outgroup members (*M* = 0.41, *SD* = 0.05) than the micro-expressions of ingroup members (*M* = 0.32, *SD* = 0.04) (see Table 2 and Figure 3). The results also showed that the duration of micro-expressions and the METT training did not moderate this ingroup disadvantage (see Table 2). In addition, the main effect of METT training and the METT training × test interaction were also significant (see Table 2). Simple effects analysis revealed that the METT training × test interaction was driven by the differences in recognition accuracies between the training condition (*M* = 0.44, *SD* = 0.04) and the control condition (*M* = 0.35, *SD* = 0.04) in the post-test of micro-expression recognition task [*F*(1,82) = 85.32, *p* < 0.001, η_p_^2^ = 0.51]. No significant differences were found between the two different conditions of METT training (training: *M* = 0.35, *SD* = 0.04; control: *M* = 0.34, *SD* = 0.03) in the pre-test of micro-expression recognition task [*F*(1,82) = 0.31, *p* = 0.58, η_p_^2^ = 0.004]. Consistent with Study 1A, the other two-way, three-way, and four-way interactions were all not significant (see Table 2).

**Table 2:** The results of mixed model ANOVA in Study 2A.

Effect	*F*	*p*	η_p_^2^
Target group	251.21	<0.001***	0.75
Duration	169.78	<0.001***	0.67
METT training	35.01	<0.001***	0.30
test	190.29	<0.001***	0.70
Target group × duration	0.07	0.79	0.001
Target group × METT training	0.21	0.65	0.002
Target group × test	0.02	0.89	<0.001
Duration × METT training	0.43	0.51	0.01
Duration × test	2.17	0.15	0.03
METT training × test	126.3	<0.001***	0.61
Target group × duration × METT training	0.001	0.98	<0.001
Target group × duration × test	0.45	0.5	0.01
Target group × METT training × test	3.21	0.08	0.04
Duration × METT training × test	2.8	0.1	0.03
Target group × duration × METT training × test	0.08	0.79	0.001

***p < 0.001.

Consistent with Study 1A, in Study 1B we also found that participants were more accurate at recognizing the subtle micro-expressions of their racial outgroups rather than the subtle micro-expressions of their racial ingroups. Taken together, the results of Study 1A and 1B suggest that there might be an ingroup disadvantage for micro-expression recognition under the situation of salient real-world groupings.

## Study 2

In Study 1, we mainly investigated the intergroup bias in micro-expression recognition by employing real-world racial groups. However, employing this type of group membership manipulation alone is not sufficient enough for researchers to completely ensure that the ingroup disadvantage found in Study 1 was not caused by some specific properties of different racial groups (e.g., the differential difficulties in recognizing micro-expressions of different racial groups). In studies of the macro-expressions, researchers have found that the recognition of macro-expression is a social categorization-based process that mere social categorization alone (i.e., using the minimal group paradigm) is sufficient to induce the ingroup advantage effect in macro-expression recognitions (Thibault et al., 2006; Young and Hugenberg, 2010; Young and Wilson, 2018). Therefore, if the ingroup disadvantage found in Study 1 was really caused by the effects of expressers’ group membership, we should also be able find the ingroup disadvantage for micro-expression recognition under the minimal group situations.

In Study 2, we further investigated that whether the nominal ingroup-outgroup distinction alone is sufficient to elicit the ingroup disadvantage in micro-expression recognition by using the minimal group paradigm. More specifically, in Study 2, participants were randomly assigned to an arbitrary group based on an ostensible personality assessment, which creates the minimal group distinction (i.e., only the label of social group is different) between the nominal ingroup members and outgroup members (e.g., Young and Hugenberg, 2010; Wu et al., 2015, 2016a, 2019; Young and Wilson, 2018; Makhanova et al., 2022). In Study 2A, we investigated whether there is an ingroup disadvantage in the recognition of intense micro-expressions under the minimal group situation. In Study 2B, we further investigated the ingroup disadvantage in the context of minimal groups by employing subtle micro-expressions. Given that the results of Study 1A and 1B showed that the micro-expression recognition training was unable to moderate the ingroup advantage in micro-expression recognition, the METT training was not employed in Study 2.

### Study 2A

#### Participants and design

The required sample size was calculated with G*Power based on the same parameters of Study 1A. A sample size of 84 participants was estimated. Finally, a total of 88 Chinese college students (44 females and 44 males; *M*_age_ = 21.66, *SD* = 1.30, aged 18–25 years; red personality group: *n* = 44; green personality group: *n* = 44) were recruited through online advertising.

A 2 (target group: ingroup, outgroup) × 2 (duration: 100 ms, 333 ms) × 2 (assigned personality type: red, green) mixed design was used. The assigned personality type was the between-subjects factor. The target group and the duration were the within-subjects factors.

#### Materials and procedure

Following previous studies (Young and Hugenberg, 2010; Wu et al., 2015, 2016a, 2019; Young and Wilson, 2018; Makhanova et al., 2022), an ostensible personality assessment was employed to create the minimal groups. The ostensible personality assessment was identical to Wu et al. (2015). The computer ostensibly assessed participants’ personality traits and then participants were informed that their scores from the personality assessment were characteristic of either a red or green personality (randomly assigned). They were further informed that each personality type has its own unique features and this experiment was specifically designed to examine how personality influences social perception. After that, participants were asked to wear a colored wristband indicative of their personality type. They were told that this wristband was employed to identify their particular personality type and were instructed to wear this wristband for the rest of experiment.

To generate the facial stimuli for Study 2A, 12 models (half males and half females; five Whites, three Asians, and four Blacks) from the BU-3DFE database were randomly selected. As for these 12 selected models, facial images of their neutral expressions and the six universal facial expressions that were rated to be “very high” in intensity were selected. Then these selected facial images were sized to 512 × 512 pixels and the background colors of these images were changed to red and green (i.e., two reprocessed images were produced for each selected facial image, one with red background, and one with green background). Finally, a total of 168 reprocessed facial images were generated and were employed as the facial stimuli for Study 2A.

**Figure 4:**
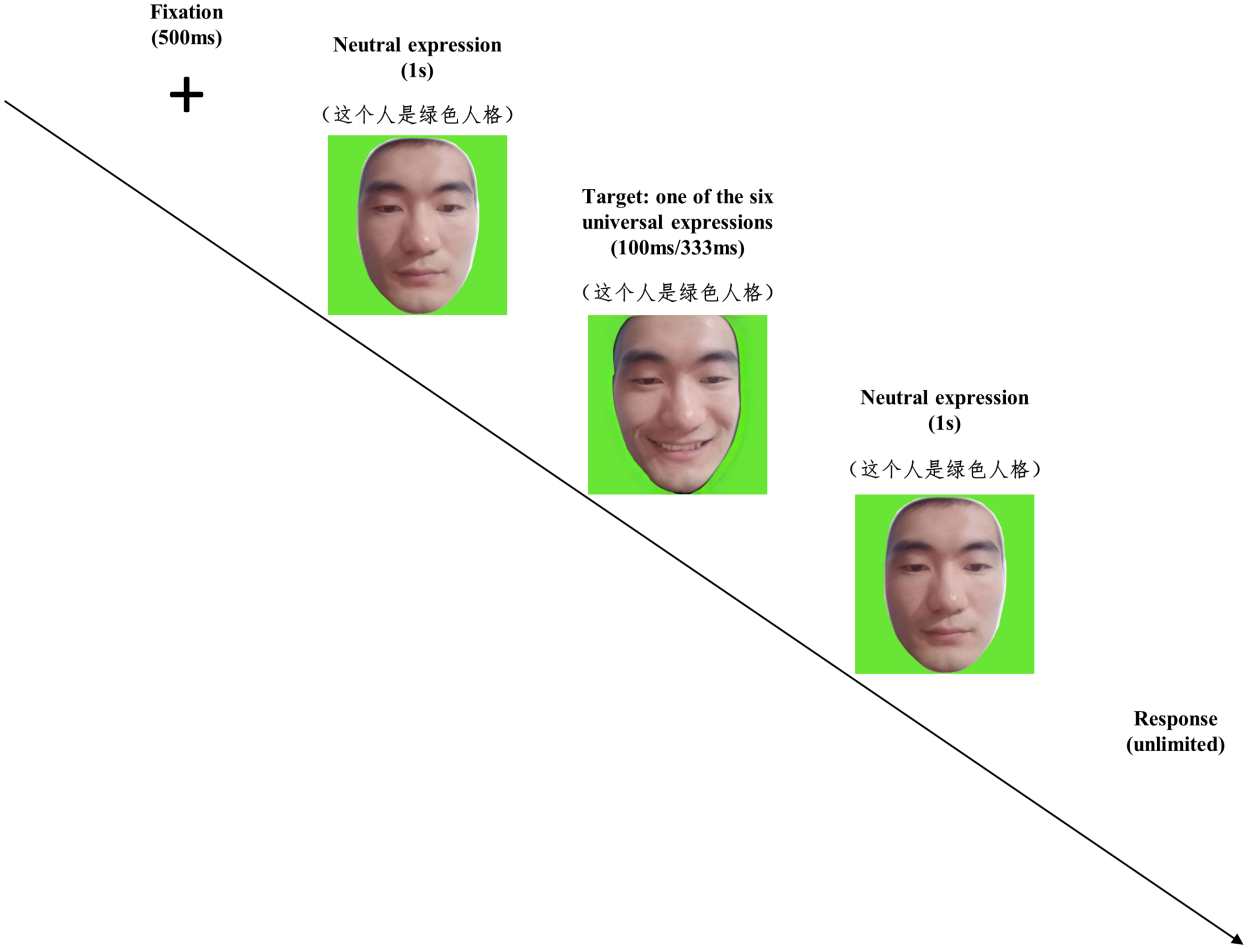
The procedure of the micro-expression recognition task in Study 1B. An example of target with green personality is presented. Note that we employ the facial images of the second author for illustration.

In Study 2A, we also employed the JACBART paradigm to present micro-expressions. A fixation cross was presented for 500 ms at first, then a reprocessed facial expression image from the facial stimuli of Study 2A were presented for 100 ms or 333 ms, which was sandwiched in between two 1 s presentation of the same expresser’s neutral face. Participants were informed before the micro-expression recognition task that the background color of the stimuli indicates the personality type of the target, and a personality tag would also appear on top of facial images to indicate the target’s personality (see Figure 4). The task of participants was to identify the facial expression just displayed as accurate as possible (by choosing one out of seven options as in Study 1A). In micro-expression recognition task of Study 2A, the 12 selected models were randomly divided into four different model groups (i.e., three models in each group, and there were at least one female model and one male model in each group). A random assignment was made between the four different presentation conditions (i.e., ingroup-100 ms, ingroup-333 ms, outgroup-100 ms, outgroup-333 ms) and the four model groups, and the six universal micro-expressions of the models were presented according to their assigned presentation conditions. The combination of the model groups and the presentation conditions was counterbalanced across participants. For each model, each micro-expression was presented only once, and the stimulus presentation was completely randomized. The colored backgrounds of facial stimuli and the personality labels displayed on the top of the facial stimuli served as the ingroup-outgroup manipulation of the targets. Therefore, half of the models were presented as having the same personality type as the participants, and the other half of models were presented as having a different personality type (see Figure 4). There was a total of 72 trials in the micro-expression recognition task of Study 2A, and the recognition accuracy was recorded for this task.

Participants were asked to take the personality assessment at first and then were instructed to complete the micro-expression recognition task. After that, participants were asked to report their assigned personality type. Finally, participants were debriefed and thanked for their participation.

#### Results and discussion

**Figure 5:**
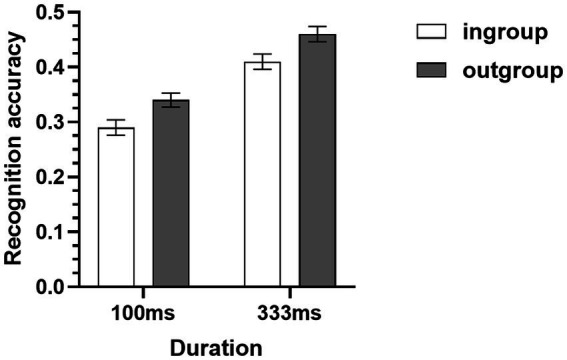
Mean recognition accuracies of micro-expressions of ingroup and outgroup members in Study 2A. Error bars represent standard errors.

All participants correctly reported their assigned personality type. The results of a 2 (target group) × 2 (duration) × 2 (assigned personality type) mixed-model ANOVA showed that the main effect of target group was significant, participants still displayed higher recognition accuracy for micro-expressions of outgroup members (*M* = 0.40, *SD* = 0.11) than the micro-expressions of ingroup members (*M* = 0.35, *SD* = 0.11) under the situation of minimal groups, *F*(1,86) = 17.00, *p* < 0.001, η_p_^2^ = 0.17 (see Figure 5). The results also showed that the duration of micro-expressions [*F*(1,86) = 0.02, *p* = 0.88, η_p_^2^ < 0.001] and the assigned personality type of participants [*F*(1,86) = 0.05, *p* = 0.82, η_p_^2^ < 0.001] did not affect this ingroup disadvantage. In addition, the main effect of assigned personality type [*F*(1,86) = 0.004, *p* = 0.95, η_p_^2^ < 0.001], the duration × assigned personality type interaction [*F*(1,86) = 0.35, *p* = 0.55, η_p_^2^ = 0.004], and the target group × duration × assigned personality type interaction [*F*(1,86) = 1.10, *p* = 0.30, η_p_^2^ = 0.01], were all not significant.

Consistent with Study 1A and 1B, Study 2A found an ingroup disadvantage in intense micro-expression recognition by using the minimal group paradigm. As a wide-accepted paradigm to study intergroup bias in the laboratory, the minimal group paradigm creates a nominal distinction between the ingroup and the outgroup while the nonverbal dialect, culture, and other social categorical distinctions are held constant (Tajfel et al., 1971; Young and Hugenberg, 2010; Wu et al., 2015, 2016a, 2019; Young and Wilson, 2018; Makhanova et al., 2022). Therefore, the results of Study 2A suggest that mere social categorization alone is sufficient to elicit ingroup disadvantage in the recognition of intense micro-expressions. In Study 2B, we further investigated the effects of expresser’s group membership on the recognition of subtle micro-expressions by employing the minimal group paradigm.

### Study 2B

#### Participants and design

The required sample size was calculated with G*Power based on the same parameters of Study 1A. A sample size of 84 participants was estimated. Finally, a total of 84 Chinese college students (42 females and 42 males; *M*_age_ = 23.81, *SD* = 2.28, aged 18–28 years; red personality group: *n* = 42; green personality group: *n* = 42) were recruited through online advertising.

A 2 (target group: ingroup, outgroup) × 2 (duration: 100 ms, 333 ms) × 2 (assigned personality type: red, green) mixed design was used. The assigned personality type was the between-subjects factor. The target group and the duration were the within-subjects factors.

#### Materials and procedure

Study 2B employed the same materials, experimental tasks, and procedure of Study 2A, except that the facial expressions in Study 1B was low in intensity. These facial expressions were also selected from the 12 models (half males and half females; six Whites and six Asians) of BU-3DFE database, but we only employed the facial expressions that were rated to be “low” in intensity.

#### Results and discussion

**Figure 6:**
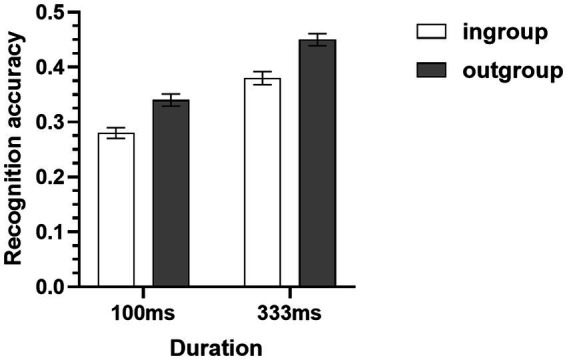
Mean recognition accuracies of micro-expressions of ingroup and outgroup members in Study 2B. Error bars represent standard errors.

All participants correctly reported their assigned personality type. The recognition accuracies of the micro-expression recognition task were subjected to a 2 (target group) × 2 (duration) × 2 (assigned personality type) mixed-model ANOVA. Consistent with Study 2A, the results showed that there was a significant main effect of target group, participants still displayed higher recognition accuracy for micro-expressions of outgroup members (*M* = 0.39, *SD* = 0.08) than the micro-expressions of ingroup members (*M* = 0.32, *SD* = 0.08) under the situation of minimal groups, *F*(1,82) = 48.12, *p* < 0.001, η_p_^2^ = 0.37 (see Figure 6). Consistent with Study 1A, this ingroup disadvantage under minimal group context was not moderated by the duration of micro-expressions [*F*(1,82) = 0.33, *p* = 0.57, η_p_^2^ = 0.004] and the assigned personality type of participants [*F*(1,82) = 0.72, *p* = 0.40, η_p_^2^ = 0.01]. The main effect of assigned personality type [*F*(1,82) = 1.94, *p* = 0.17, η_p_^2^ = 0.02], the duration × assigned personality type interaction [*F*(1,82) = 0.06, *p* = 0.81, η_p_^2^ = 0.001], and the target group × duration × assigned personality type interaction [*F*(1,82) = 1.07, *p* = 0.31, η_p_^2^ = 0.01], also were all not significant.

Therefore, the results of Study 2B showed that there was also an ingroup disadvantage in the subtle micro-expression recognition under the minimal group paradigm. Taken together, the results of Study 2A and 2B suggest that the nominal ingroup-outgroup distinction alone is sufficient to elicit the ingroup disadvantage for the recognition of micro-expressions.

## General discussion

Previous findings on macro-expressions revealed a widespread ingroup advantage in emotion perception, as individuals tend to be more accurate when recognizing the macro-expressions produced by members of their ingroup rather than those produced by the outgroup members (Elfenbein and Ambady, 2002; Thibault et al., 2006; Elfenbein et al., 2007; Young and Hugenberg, 2010; Huang and Han, 2014; Tuminello and Davidson, 2011; Zhang et al., 2015; Gamond et al., 2017; Young and Wilson, 2018; Friesen et al., 2019; Kommattam et al., 2019; Handley et al., 2021; Fang et al., 2022; Hess et al., 2022). Contrary to the researches on macro-expression recognition, the present study is the first to demonstrate that there is no ingroup advantage but an ingroup disadvantage for micro-expression recognitions. Specifically, in Study 1A and 1B, we found that participants were more accurate at recognizing the intense and subtle micro-expressions of their racial outgroups, and such an ingroup disadvantage was not moderated by the duration of micro-expressions and the training experience of participants. In Study 2A and 2B, we further found that mere social categorization alone was sufficient to elicit the ingroup disadvantage for the recognition of intense and subtle micro-expressions, and such an effect was also unaffected by the duration of micro-expressions. Moreover, the results indicate that the ingroup disadvantages in micro-expression recognition cannot be explained by differences in skin colors or textures between expressers of different social groups, since these differences were already controlled by the procedures of image normalization (in Study 1A and 1B) or by the randomization and counterbalance of stimulus (in Study 2A and 2B). The results also showed that the ingroup disadvantage was not caused by the differential difficulties in the recognition of macro-and micro-expressions of different social groups since this factor was already controlled by matching the recognition accuracy of the macro-expressions of different social groups (in Study 1A and 1B) or by taking randomization and counterbalance measures (in Study 2A and 2B). Therefore, these results are consistent with our hypothesis which suggest that there might be a robust ingroup disadvantage in the recognition of micro-expressions and there are fundamental differences in the psychological mechanisms for the recognition of micro-expressions and macro-expressions. The results further suggest that we may need to control the intergroup bias when we are building micro-expression databases (e.g., the coders of database should be recruited from diverse social backgrounds to avoid the potential coding bias; Yan et al., 2014; Yap et al., 2020; Li et al., 2022).

Why is there an ingroup disadvantage for micro-expression recognition? Previous studies on macro-expression recognition suggest that the ingroup advantage in emotion perception stems from a multitude of coacting factors, including both greater familiarity with ingroup expressive norms and dialects and greater motivation to attend to and process ingroup emotional signals (Thibault et al., 2006; Elfenbein et al., 2007; Young and Hugenberg, 2010; Elfenbein, 2015; Young and Wilson, 2018; Hess et al., 2022). In the present research, we found clear evidence that mere ingroup-outgroup distinctions alone can reliably elicit ingroup disadvantage in micro-expression recognition, which suggests that the intergroup bias in micro-expression recognition is a social-categorization based process and it is caused by the motivated differential processing of micro-expressions of ingroup members. In fact, previous studies have suggested that such differential processing of ingroup micro-expressions may stem from the differential effects of endogenous oxytocin and facial mimicry on ingroup members. That is, the endogenous neuropeptide of oxytocin and the facial mimicry generated during emotion perception are more likely to disrupt the recognition of micro-expressions of ingroup members (De Dreu et al., 2011; Stallen et al., 2012; Hess and Fischer, 2014; Wu et al., 2016a, 2022; McClung et al., 2018; Zeng et al., 2018; Hess et al., 2022). Given the close association between oxytocin and facial mimicry (i.e., oxytocin may facilitate the production of facial mimicry; Korb et al., 2016; Pavarini et al., 2019), the previous studies also suggest that the oxytocin system may be the neurophysiological basis for the ingroup disadvantage effect in micro-expression recognitions. However, we should note that the results of the present study cannot rule out the possibility that the nonverbal dialects have also affected the recognition of micro-expressions since we had also found the ingroup disadvantage under racial group situations (in Study 1A and 1B) in which the nonverbal dialect differences were not controlled. Researchers need to recruit participants from more diverse culture backgrounds (e.g., White participants) and employ instructed emotional faces (e.g., Tsikandilakis et al., 2021) to explore this issue in the future.

Contrary to the universal tendency of ingroup favoritism found across cultures (e.g., Tajfel et al., 1971; Hewstone et al., 2002; Ji et al., 2019; Makhanova et al., 2022), a counterintuitive phenomenon of ingroup derogation (i.e., the tendency to favor members of one’s outgroup over members of one’s ingroup) has also been reported (mainly in the East Asian cultures; Ma-Kellams et al., 2011; Zhao et al., 2012; Wu et al., 2015, 2016b, 2019). For example, it was reported that the Chinese participants tended to associate Westerners with more positive characteristics than members of their own ethnic group (Ma-Kellams et al., 2011). Moreover, it was found that Chinese participants also rated outgroup members’ faces and names as more beautiful and better (Zhao et al., 2012; Wu et al., 2016b), and they also displayed more cooperation tendency toward the members of outgroup under minimal group situations (Wu et al., 2015, 2016b, 2019). Given that in the present research we also exclusively focused on Chinese participants, these previous studies suggest that the ingroup disadvantage found in the present research can be viewed as a manifestation of ingroup derogation at the perceptual level. In fact, previous studies on macro-expressions have also reported that the ingroup advantage effect tend to be unstable for Chinese participants (e.g., Beaupré and Hess, 2006; Mondillon et al., 2007; Prado et al., 2014; Zhang et al., 2015). However, it should be noted that the adaptation account of ingroup favoritism (Navarrete and Fessler, 2006; Van Vugt and Park, 2009; Fu et al., 2012) may also offer the explanation for the ingroup disadvantage phenomenon found in the present study. That is, individuals harbor ingroup favoritism attitudes because it is the ingroup members who offer us the important chances of cooperation and the necessary protections against intergroup violence and disease threats (Navarrete and Fessler, 2006; Van Vugt and Park, 2009; Fu et al., 2012). Therefore, given the close association between the production of micro-expressions and deception or hiding of emotions (Ekman, 2003, 2009; Matsumoto and Hwang, 2011, 2018; Yan et al., 2013), having an ingroup disadvantage in micro-expression perception may still be adaptive for individuals since moderately inhibiting the processing of information associated with one’s untrustworthiness may facilitate the valuable ingroup cohesions that are important for one’s survival (Pfundmair et al., 2017; Wu et al., 2022). Nonetheless, the current evidence obtained in the present research is not sufficient for us to discriminate between these two possibilities (i.e., the culture-specific ingroup derogation account and the universal adaptive ingroup favoritism account). Cross-cultural researches, such as conducting researches in Western cultures, are needed to investigate the universality of ingroup disadvantage in micro-expression recognition in the future.

Will the effects of expressers’ group membership be consistent for the recognition of micro-expressions from different emotion categories? Given the main purpose of the current research was to investigate the effects of group membership on the overall micro-expression recognition performance, in the present study, we had only employed three trials for each category of micro-expressions under each condition. Such an experimental design would greatly undermine the statistical power for the analysis of the interaction of expressers’ group membership and emotion category (Wu et al., 2016a; Zeng et al., 2018). Therefore, we simply dropped the factor of emotion category in our analysis. Previous studies employing similar trial numbers in micro-expression recognition also did not include this factor into consideration or simply found the nonsignificant interactions (Wu et al., 2016a; Zeng et al., 2018). However, it should be noted that one recent study employing more trials (12 trials) for each category of micro-expression did find the significant interaction between the intended experimental manipulation (administration of oxytocin) and the emotion category (Wu et al., 2022). In addition, it also should be note that employing too less trials for a specific category of micro-expressions prevents the researchers from analyzing the recognition error patterns of ingroup and outgroup micro-expressions by using confusion matrix (Shen et al., 2012). Researchers need to employ more emotion-specific experimental design (e.g., focusing on two or three representative kinds of micro-expressions; e.g., Zhang et al., 2020) and more trials (e.g., Shen et al., 2012) to solve these issues in the future.

In the present research, the JACBART paradigm was employed to present micro-expressions. As a well-accepted method to present micro-expressions in the laboratory (e.g., Hurley, 2012; Wu et al., 2016a, 2022; Demetrioff et al., 2017; Zhang et al., 2020; Döllinger et al., 2021; Fan et al., 2022), this paradigm enables effectively manipulation of the core features of micro-expression (e.g., the intensity or duration of micro-expressions) without incurring interference (e.g., the head movement of models). However, it should be noted that besides its advantage in internal validity, JACBART paradigm does have some limitations in its ecological validity: It only utilizes three still images to synthesize the facial dynamics of micro-expressions (Matsumoto et al., 2000), which is significantly different from the natural micro-expression in realistic settings (Yan et al., 2013; Li et al., 2022; Wu et al., 2022). Although the previous research suggests that the recognition of JACBART synthesized micro-expressions is very similar to the recognition of natural micro-expressions (Wu et al., 2022), researchers in the future still need to address this ecological issue by employing more naturalistic dataset (e.g., Yan et al., 2014; Yap et al., 2020; Li et al., 2022). In addition, it also should be noted that although the results of the present study suggest that the ingroup disadvantage in micro-expression recognition cannot be moderated by the duration of micro-expression or by the training experience of the perceivers, it is still possible that such results might be caused by the specific setting of METT training or by the specific setting of durations in the present research. For example, by repetitiously investigating the effects of facial feedback under four different settings of duration in the experiment, one recent study eventually found out that enhancing facial mimicry may only affect the recognition accuracy of intense micro-expressions under long duration conditions (e.g., 450 ms; Zeng et al., 2018). Therefore, researchers may still need employ more diverse settings of durations (e.g., Shen et al., 2012) and more intensive micro-expression recognition trainings (e.g., Hurley, 2012) to further test the boundary condition of ingroup disadvantage effect in the future.

## Conclusion

Contrary to the widespread ingroup advantage found in macro-expression recognition, the current findings indicate that there might be a robust ingroup disadvantage in micro-expression recognition instead, which further suggests that there are fundamental differences in the psychological mechanism for the recognition of micro-expressions and macro-expressions. These findings may facilitate the creation of more valid micro-expression databases and more effective micro-expression recognition training tools.

## Data availability statement

The raw data supporting the conclusions of this article will be made available by the authors, without undue reservation.

## Ethics statement

The studies involving human participants were reviewed and approved by Research Ethics Committee of Hunan Normal University. The patients/participants provided their written informed consent to participate in this study.

## Author contributions

QW designed the studies. YX, YL, XL, and ZZ recruited the participants and collected the data. QW and KP wrote the first draft of the manuscript. All authors participated in analyzing the experimental data, revising the manuscript, and approved the submitted version.

## Funding

This work was supported by the Outstanding Young Scientific Research Project of Hunan Provincial Department of Education (No. 19B361).

## Conflict of interest

The authors declare that the research was conducted in the absence of any commercial or financial relationships that could be construed as a potential conflict of interest.

## Publisher’s note

All claims expressed in this article are solely those of the authors and do not necessarily represent those of their affiliated organizations, or those of the publisher, the editors and the reviewers. Any product that may be evaluated in this article, or claim that may be made by its manufacturer, is not guaranteed or endorsed by the publisher.

## Footnotes

^1^Study 1A has been presented at the conference of FG019 as an oral presentation (Xie et al., 2019).
